# Mating alters gene expression patterns in *Drosophila melanogaster *male heads

**DOI:** 10.1186/1471-2164-11-558

**Published:** 2010-10-11

**Authors:** Lisa L Ellis, Ginger E Carney

**Affiliations:** 1 Department of Biology, Texas A&M University, College Station, TX USA 77843-3258; 2 Current Address: Department of Entomology, Texas A&M University, College Station, TX USA 77843-2475

## Abstract

**Background:**

Behavior is a complex process resulting from the integration of genetic and environmental information. *Drosophila melanogaster *rely on multiple sensory modalities for reproductive success, and mating causes physiological changes in both sexes that affect reproductive output or behavior. Some of these effects are likely mediated by changes in gene expression. Courtship and mating alter female transcript profiles, but it is not known how mating affects male gene expression.

**Results:**

We used *Drosophila *genome arrays to identify changes in gene expression profiles that occur in mated male heads. Forty-seven genes differed between mated and control heads 2 hrs post mating. Many mating-responsive genes are highly expressed in non-neural head tissues, including an adipose tissue called the fat body. One fat body-enriched gene, *female-specific independent of transformer *(*fit*), is a downstream target of the somatic sex-determination hierarchy, a genetic pathway that regulates *Drosophila* reproductive behaviors as well as expression of some fat-expressed genes; three other mating-responsive loci are also downstream components of this pathway. Another mating-responsive gene expressed in fat, *Juvenile hormone esterase *(*Jhe*), is necessary for robust male courtship behavior and mating success.

**Conclusions:**

Our study demonstrates that mating causes changes in male head gene expression profiles and supports an increasing body of work implicating adipose signaling in behavior modulation. Since several mating-induced genes are sex-determination hierarchy target genes, additional mating-responsive loci may be downstream components of this pathway as well.

## Background

Behavior involves the perception and processing of sensory information into a signaling cascade that mediates physiological and motor outputs. This complex process is influenced by an organism's environment, genetic make-up and nervous system function. Social interactions influence an organism's behavior [1-5], and these behavioral changes are associated with alterations in morphology [6-9] and gene expression [6,10-17]. However, the mechanisms mediating the changes are unclear. As we work to understand responses to behavior at the transcript level, we can clarify the regulatory and intracellular processes governing nervous system function and behavior.

Therefore, we are studying reproductive behaviors in the genetically tractable *Drosophila melanogaster*, which exhibit stereotypical mating behaviors [reviewed in [18,19]] regulated by genetics [reviewed in [20,21]] and social interactions [[1,22,23]; reviewed in [19,24,25]]. The sex-determination gene hierarchy is the major regulator of *Drosophila *reproduction [reviewed in [26,27]]. Components of this pathway affect sexually dimorphic development, including the neural circuitries necessary for sex-specific courtship behaviors [28-32]. However, the behavioral functions of only a few of the downstream target genes of the hierarchy are known [33-43].

Although the potential for performing courtship behavior is under genetic control, experience with other individuals alters behavior, particularly in the context of courtship learning [19,24,25]. During courtship and mating, the male is inundated with sensory information that must be interpreted so that the appropriate signals are sent throughout the body for a successful mating. Therefore, it is reasonable to expect that a more experienced male would be better at performing some aspect of courtship to improve his mating success. In support of this idea, *Drosophila *males experienced at courting females initiate courtship toward novel, receptive females more quickly than do inexperienced males [44,45]. In a natural setting where many flies are competing for mates, rapid courtship initiation may give an experienced male a competitive advantage that increases his mating success. Simply observing courtship and mating behavior of other flies is not sufficient to decrease the male's own mating latency, indicating that this learning behavior requires active participation [45]. It is possible that changes in courting and mated male gene expression underlie this decreased courtship latency in subsequent interactions.

By combining behavioral assays with microarray technology, it is possible to assess behaviorally-responsive gene expression changes on a genome-wide scale [12,22,46-51] to find loci regulating or regulated by behavior, including sex-determination hierarchy target genes. Prior work in our lab demonstrated that males rapidly alter gene expression at the whole-animal level during courtship [12,22]. Next, we focused on changes occurring in the male head as a result of mating since these changes likely affect function of the nervous system and other reproductively important tissues to promote reproductive success. Our study demonstrates that courtship culminating in mating affects gene expression patterns in male heads and that many of the gene products are expressed in non-neural adipose tissue that may play an important modulatory role in neural function and behavior.

## Results and Discussion

### Mating causes expression changes in male heads

Gene expression levels change rapidly as males court females [12,22]. To determine the effects of courtship culminating in mating on male gene expression, we compared transcriptional profiles of males that mated with a female to those that were not presented with a female (control). Labeled samples from control and treatment groups were hybridized to *Drosophila *Genome 2.0 Arrays (Affymetrix, Santa Clara, CA, USA), which are based on the Flybase 3.1 annotation, targeting nearly 18,500 transcripts.

**Figure 1:**
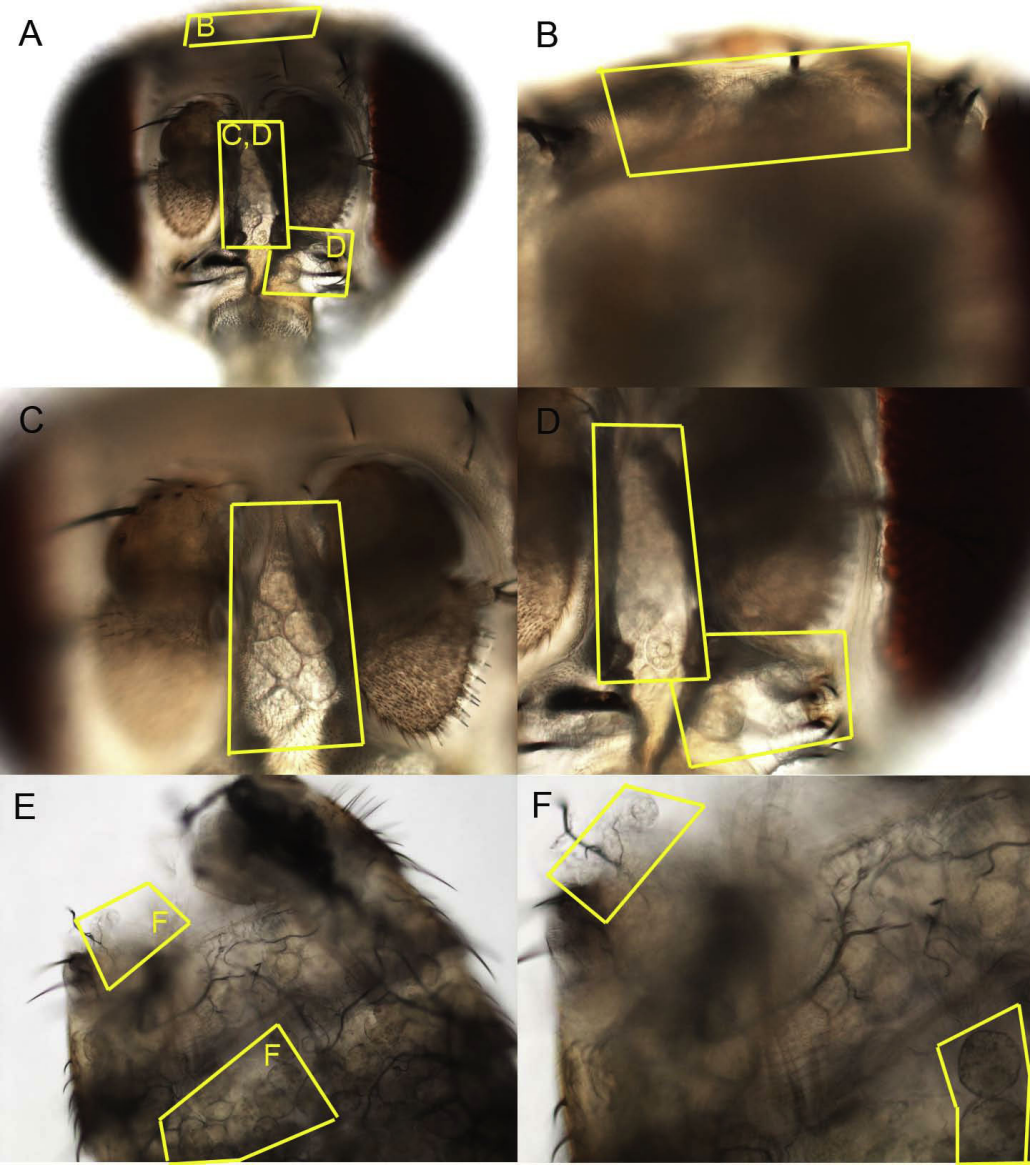
**Fat body tissue in the adult male**. Low magnification image (10×) from the front of an adult male head (A) or dorsal abdominal cuticle (E). Boxed areas indicate adipose tissue magnified at 20× in 3 areas of the head (B-D) and 2 areas along the abdominal cuticle (F).

In the current study we focused on head expression, rather than whole body expression [12,22], to identify gene expression changes in the nervous system and other tissues within the head (such as sensory systems and fat body) that likely modulate reproduction. We isolated male heads (rather than dissecting out the brains) since accumulating evidence from our lab [[12,22]; L.L. Ellis and G.E. Carney, unpublished results] as well as from other published studies [[37,39,40,43]; reviewed in [52]] indicates that head tissues, such as the fat body surrounding the brain (Fig. 1), likely also have important modulatory functions in behavior. To have the potential to identify gene expression changes in these tissues as well, we elected to assay the entire male head for alterations in gene expression patterns in response to mating.

We used five algorithms to extract expression values from each array and performed paired *t*-test comparisons between the expression values derived from mated male head arrays and control male head arrays. Using this strategy we identified 47 mating-responsive genes (See Methods). Two hours after mating with a female, males significantly up regulated 25 genes (Table 1) and down regulated 22 genes (Table 2). Such changes are not likely to be activity-dependent since control males had locomotor levels similar to males that courted females (two-tailed *t*-test, p > 0.05).

**Table 1:** Candidate genes up regulated 2 hrs after mating

Gene identifier	Gene name	Avg. fold change	GO Molecular function	GO Biological process
*CG2163*	*polyA-binding protein II *(*Pabp2*)	1.4	Poly(A) binding	mRNA polyadenylation
*CG4288*		1.28	High affinity inorganic phosphate: sodium symporter activity	Transport
*CG4501*	*bubblegum *(*bgm*)	1.38	Long-chain-fatty acid-CoA ligase activity	Long-chain fatty acid metabolic process
*CG4825*	*Phosphatidyl-serine synthase*	1.22	CDP-diacylglycerol-serine O-phosphatidyltrans-ferase activity	Phosphatidyl-serine biosynthetic process
*CG5527*		1.23	Endothelin-converting enzyme activity	Proteolysis
*CG5618*		1.14	Dipeptidyl-peptidase III activity	Proteolysis
*CG6188*		1.64	Glycine N-methyltransferase activity	Unknown
*CG6342*	*Iron regulatory protein 1B *(*Irp-1B*)	1.26	Iron ion binding	Regulation of translational initiation by iron
*CG8425*	*Juvenile hormone esterase *(*Jhe*)	1.86	Juvenile-hormone esterase activity	Juvenile hormone catabolic process
*CG8449*		1.28	Rab GTPase activator activity	Regulation of Rab GTPase activity
*CG9989*		1.52	Endonuclease activity	Unknown
*CG11765*	*Peroxiredoxin 2540 *(*Prx2540-2*)	1.2	Antioxidant activity	Unknown
*CG12116*		1.22	Sepiapterin reductase activity	Metabolic process
*CG13360*		1.28	Unknown	Unknown
*CG13607*		1.23	Unknown	Unknown
*CG13965*		1.35	Unknown	Unknown
				
*CG16772*		1.5	Unknown	Unknown
*CG16901*	*squid *(*sqd*)	1.25	mRNA binding	Oocyte axis determination
*CG17364*		1.67	GTP binding	Microtubule-based process
*CG17820*	*female-specific independent of transformer *(*fit*)	1.4	Unknown	Unknown
*CG18262*		1.3	Zinc ion binding	Unknown
*CG30026*		1.42	Unknown	Unknown
*CG30095*		1.86	Oxidoreductase activity	Metabolic process
*CG30084*	*Z band alternatively spliced PDZ-motif protein 52 *(*Zasp52*)	1.38	Protein binding	Unknown
*CG33486*	*asparagine synthetase*	1.28	Asparagine synthetase (glutamine-hydrolyzing) activity	Asparagine biosynthetic process

Twenty-five genes are significantly (p < 0.001) up regulated in male heads 2 hrs after mating when compared to control male heads.

**Table 2:** Candidate genes down regulated 2 hrs after mating

Gene identifier	Gene name	Avg. fold change	GO Molecular function	GO Biological process
*CG1897*	*Drop *(*Dr*)	-1.5	DNA binding	Central nervous system development
*CG2505*	*α-Esterase-2 *(*α-Est2*)	-1.3	Carboxylesterase activity	Unknown
*CG3200*	*Rhythmically expressed gene 2 *(*Reg-2*)	-1.27	Phosphoglycolate phosphatase activity	Metabolic process
*CG3926*	*Serine pyruvate aminotrans-ferase *(*Spat*)	-1.34	Serine-pyruvate transamine activity	Glyoxylate catabolic process
*CG4105*	*Cytochrome P450-4e3 *(*Cyp4e3*)	-1.3	Electron carrier activity	Unknown
*CG5840*		-1.34	Pyrroline-5-carboxylate reductase activity	Proline biosynthetic process
*CG6806*	*Larval serum protein 2 *(*Lsp2*)	-1.28	Nutrient reservoir activity	Transport
*CG7224*		-1.16	Unknown	Unknown
*CG7390*	*senescence marker protein-30 *(*smp-30*)	-1.36	Unknown	Unknown
*CG8112*		-1.42	Sterol O-acyltransferase activity	Unknown
*CG8846*	*Thor*	-1.26	Eukaryotic initation factor 4E binding	Immune response
*CG9416*		-1.25	Sequence-specific DNA binding	Regulation of transcription
*CG9733*		-1.6	Trypsin activity	Proteolysis
*CG11909*	*target of brain insulin *(*tobi*)	-1.42	α-glucosidase activity	Carbohydrate metabolic process
*CG11919*		-1.36	ATP binding	Peroxisome organization and biogenesis
*CG16898*		-1.68	Unknown	Unknown
*CG18003*		-1.36	Glycolate oxidase activity	Metabolic process
*CG30489*	*Cyp12d1-p*	-1.3	Electron carrier activity	Unknown
*CG31075*		-1.26	Aldehyde dehydrogenase (NAD) activity	Pyruvate metabolic process
*CG31628*	*adenosine 3 *(*ade3*)	-1.28	Phosphoribo-sylamine-glycine ligase activity	Purine base biosynthetic process
*CG31689*		-1.25	ATPase activity	Unknown
*CG33462*		-4.08	Trypsin activity	Proteolysis

Average fold changes, molecular functions and biological processes are shown for 22 genes that are significantly (p < 0.001) down regulated in male heads 2 hrs after mating.

### Verification of microarray results by independent qPCR

To confirm the microarray results, we performed qPCR analysis on independently collected mated and control male head RNA samples. We tested a subset of genes whose expression levels changed significantly in mated male heads compared to control male heads. Eight out of 10 up-regulated genes and 2 out of 3 down-regulated genes had the expected directional change (Table 3). We did not verify up regulation of *CG4825 *and *fit *or down regulation of *CG8112 *by qPCR. However, increased *fit *expression in the fat body lining the brain was confirmed by *in situ *hybridization (see below).

**Table 3:** Confirmation of microarray results by qPCR

Gene identifier	Gene symbol	Microarray Fold change	qPCR Relative fold change ± SEM	Avg. relative expression level in control male heads ± SEM	Avg. relative expression level in mated male heads ± SEM
*CG5618*		1.14	2.02 ± 0.49*	0.36 ± 0.09	0.74 ± 0.18
*CG6188*		1.64	1.94 ± 0.26*	2.25 ± 0.42	4.38 ± 0.58
*CG8449*		1.28	1.35 ± 0.15*	1.24 ± 0.26	1.68 ± 0.18
*CG16772*		1.5	4.07 ± 1.55	6.86 ± 1.72	27.94 ± 10.61
*CG30026*		1.42	2.23 ± 0.35*	4.36 ± 0.84	9.74 ± 1.53
*CG4501*	*bgm*	1.38	4.47 ± 1.11*	1.42 ± 0.31	6.37 ± 1.57
*CG6342*	*Irp-1B*	1.26	1.42 ± 0.12	1.09 ± 0.18	1.55 ± 0.13
*CG11765*	*Prx2540-2*	1.2	1.23 ± 0.12*	0.47 ± 0.08	0.58 ± 0.06
*CG2505*	*αEst2*	-1.3	-1.26 ± 0.15	2.84 ± 0.59	2.25 ± 0.42
*CG7390*	*smp-30*	-1.36	-1.69 ± 0.16*	1.28 ± 0.45	0.77 ± 0.2

* Indicates a significant (p < 0.05) difference between the average relative expression level in control male heads and mated male heads. SEM = Standard error of the mean.

### Expression of candidate genes is not restricted to the brain

We hypothesized that examining gene expression in head tissue instead of whole bodies would uncover genes that function in reproduction by regulating nervous system signaling. This could be via direct effects on neural gene expression or by effects on other tissues in the head that receive or respond to courtship and mating signals. We found that expression of many mating-responsive genes is enriched in the head but not the brain (Table 4) [53], indicating expression occurs outside of the brain. While some of the genes are expressed in the eye, others appear enriched in tissues other than the brain and eye.

**Table 4:** Candidate genes are enriched in head tissue other than the brain, including adult adipose tissue

	Total no. of genes	Head	Brain	Eye	Fat body
Up regulated	25	18	4	9	16
Down regulated	22	20	2	12	18

Data was compiled from FlyAtlas [53].

**Figure 2:**
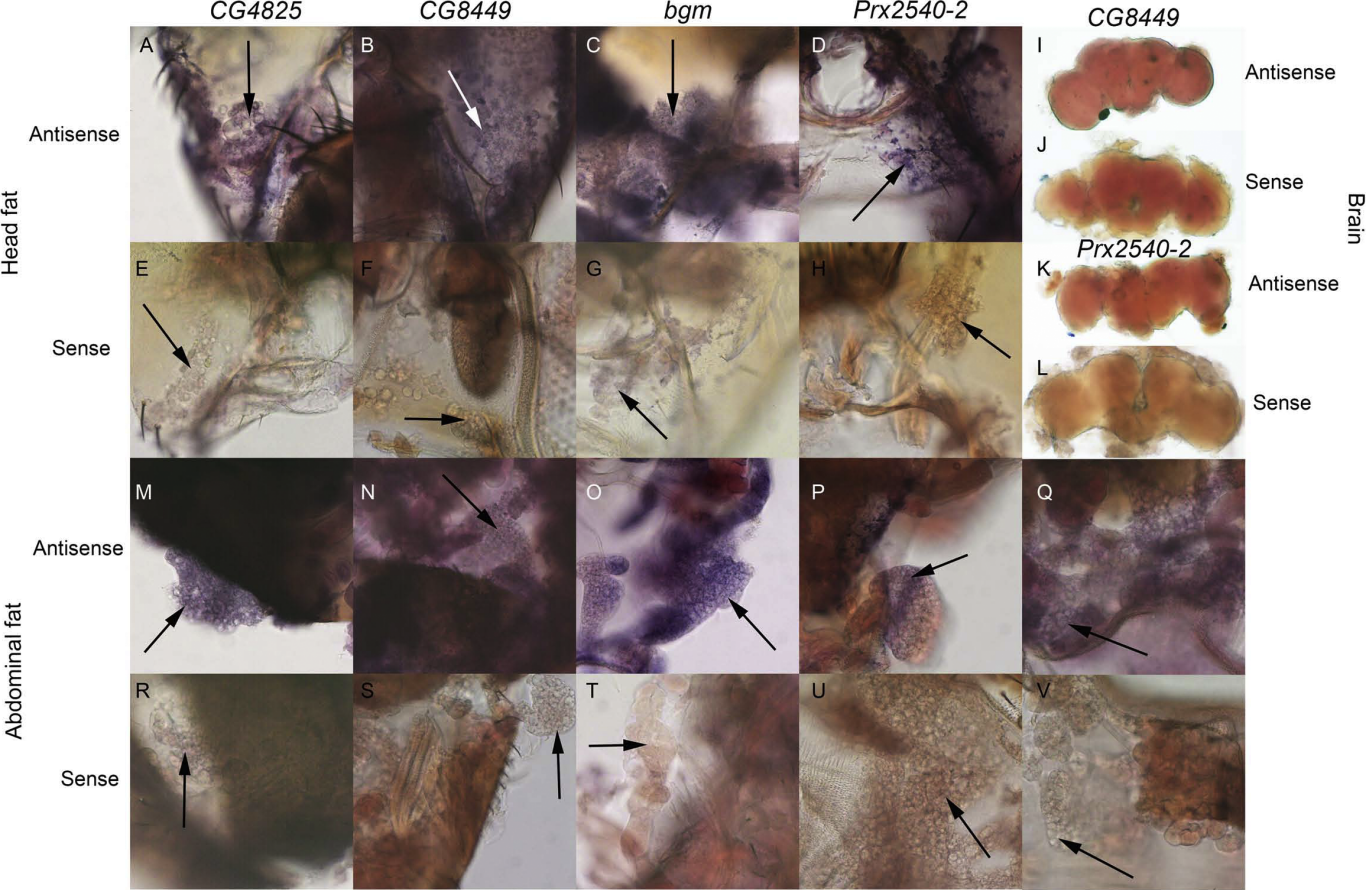
**Candidate genes are expressed in fat tissue**. Antisense (A-D,I,K,M-Q) or sense (E-H,J,L,R-V) RNA probes were designed to cDNA clones for *CG4825 *(A,E,M,R), *CG8449 *(B,F,I,J,N,S), *bgm *(C,G,O,T), *Prx2540-2 *(D,H,K,L,P,U), and *CG13360 *(Q,V). *In situ *hybridization to whole-mount tissue showed candidate gene expression in male *CS *fat body tissue (arrows) present on head (A-H) and abdominal (M-V) cuticle. Purple reactivity indicates presence of transcripts. Brains (I-L) showed light pink background staining but lacked detectable expression of either *CG8449 *(I,J) or *Prx2540-2 *(K,L).

One possibility is that they are expressed in an adipose tissue called the fat body that lines the head cavity surrounding the brain (Fig. 1a-d) and is implicated in courtship behavior modulation [[37,39,40,43]; reviewed in [52]]. Data showing that mating-responsive genes enriched in the head are also enriched in the adult fat body (Table 4) [53] support this hypothesis. *In situ *hybridization confirmed that several mating-responsive loci (*CG13360*, *bubblegum *(*bgm*), *Prx2540-2*, *CG8449 *and *CG4825*) are expressed in male fat body tissue (Fig. 2).

FlyAtlas data indicate that the fat-expressed genes *CG13360*, *bgm*, and *Prx2540-2 *are expressed at very low levels in brains, while *CG8449 *and *CG4825 *are expressed at low to moderate levels in the brain [53]. By *in situ *we did not detect brain expression of these five transcripts (Fig. 2 and data not shown), although we cannot rule out the possibility that low levels of message are present.

**Figure 3:**
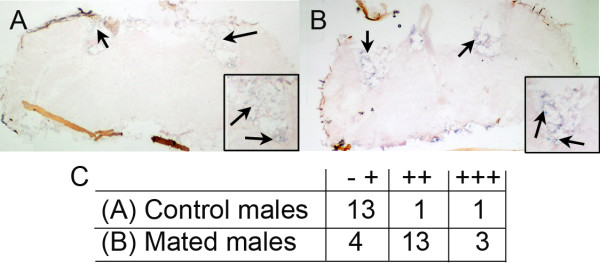
***fit *expression in the fat body is increased after courtship culminating in mating**. *In situ *hybridization was performed on cryosectioned male heads and confirmed that *fit *transcript levels were up regulated in adipose tissue (arrows) of mated males (panel B) compared to control males (panel A) as indicted by increased intensity of purple staining in mated male fat body. Insets in panels A and B show magnified views of head fat. Qualitative assessment of signal intensity in both treatment groups indicates that *fit *expression increased in mated male heads (panel C).

To test the hypothesis that mating-induced changes in gene expression occur in the fat body, we compared *fit *expression in the heads of mated and control heads. *fit *expression increased in the adipose tissue surrounding the male brain after courtship culminating in mating (Fig. 3).

### Juvenile hormone esterases are important for male reproductive behaviors

**Figure 4:**
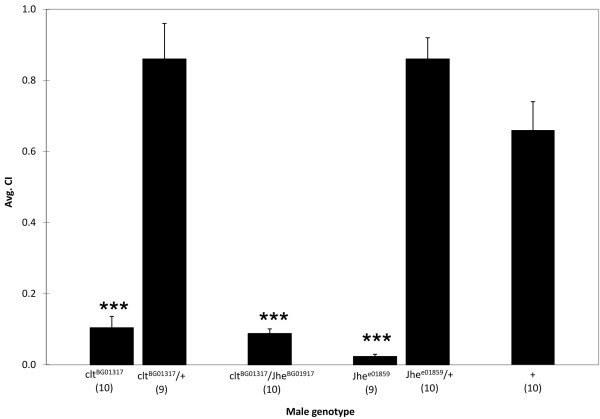
***Jhe *and *clt *mutants reduce courtship toward females**. Mutant males homozygous for P-element insertions in *Jhe *or *clt *showed reduced courtship (***p < 0.001) under red light compared to sibling heterozygous and wild-type controls. *Jhe^e01859 ^+/+ clt^BG01317 ^*mutant males showed significant reductions in courtship compared to either heterozygote or the wild-type control. (N) reflects sample size. Error bars are SEM.

We hypothesized that if a gene is up regulated after mating, that gene likely affects some aspect of reproductive behavior. Therefore, we assayed the percentage of time a male spent courting a female, known as the courtship index (CI), of candidate mating-responsive gene mutants. A *Jhe *P-element insertion, *Jhe^e01859^*, resulted in significantly reduced CI values in homozygous mutant males compared to heterozygous or wild-type controls (Fig. 4). Heterozygous males showed similar courtship activity compared to wild-type males, ruling out heterozygous effects on CI levels. Though *Jhe *males court females less vigorously, they perform standard courtship steps, eventually culminating in copulation.

In addition to *Jhe *there are three other candidate juvenile hormone esterase genes in the *Drosophila* genome [54]. One of the genes, *cricklet *(*clt*), also had an available P-element insertion, so we tested *clt^BG01317 ^*mutants to see if they had a similar phenotype to *Jhe *mutants. We found that *clt *mutants also have decreased CIs relative to controls (Fig. 4). There is also a strong genetic interaction between *Jhe *and *clt*. Transheterozygous mutant males had significantly reduced courtship compared to controls (Fig. 4).

**Figure 5:**
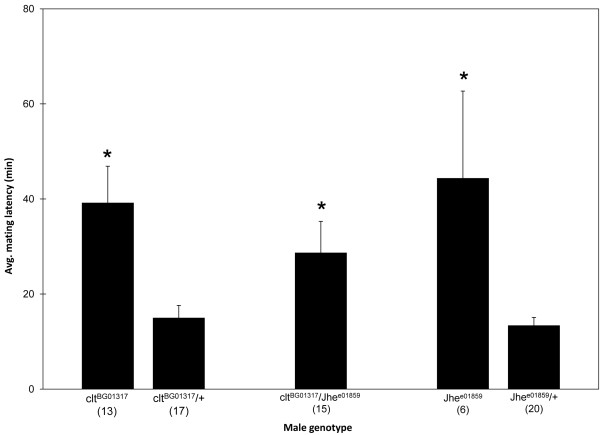
**Mating latency is increased in *Jhe *and *clt *mutants**. Under red light conditions, homozygous and transheterozygous mutant males had significantly (ANOVA p < 0.01, Tukey's *p < 0.05) increased mating latencies toward *CS *virgin females regardless of the mating bout (1^st^, 2^nd^, or 3^rd^); therefore, overall average mating latencies are shown. (N) reflects sample size. Error bars are SEM.

**Figure 6:**
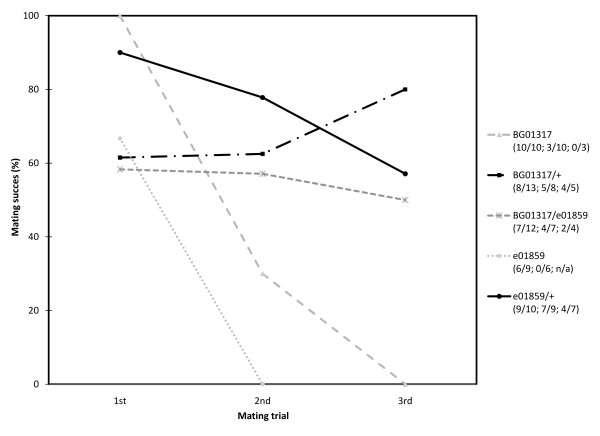
**Mating success decreases in *Jhe *and *clt *mutants**. *Jhe/+ *and *clt*/+ control males successfully mated with 3 females in succession, while experimental *Jhe *and *clt *mutant males significantly (Binary Logistic Regression, genotype p < 0.01, trial p < 0.0001, interaction p < 0.0001) decreased their mating success with the 2^nd ^and 3^rd ^females. No. of successful matings/Total no. of pairings for 1^st^; 2^nd^; 3^rd ^trials are shown.

We predicted that mating-responsive loci would function to prime the male for subsequent mating encounters by regulating courtship or mating latency and duration. Therefore, we predicted that decreasing *Jhe*, which was up regulated in mated male heads, would increase courtship or mating latency. To test this hypothesis we examined the courtship and mating kinetics of mutants for *Jhe *and the related esterase *clt*. Courtship latency (time to initiation of courtship) did not differ among mutants and controls. Though *Jhe *and *clt *males mated with females, they had a significant (p < 0.05) increase in mating latency (Fig. 5) (ANOVA, genotype p < 0.05, trial p > 0.05), while mating duration was unaffected. The increased mating latency was not dependent on the mating trial (1^st^, 2^nd ^or 3^rd^). However, as we increased the number of mating attempts, the mating success (as measured by the act of copulation) of *Jhe *and *clt *mutant males was significantly reduced (Fig. 6) (Binary Logistic Regression, genotype p < 0.01, trial p < 0.0001, interaction p < 0.0001) compared to heterozygous controls. Since we ruled out a heterozygous effect on CI values we did not test for heterozygous effects on mating latency or mating success. Females mated to *Jhe *or *clt *mutant males laid equivalent numbers of eggs regardless of the mating trial and day of egg laying (ANOVA, genotype p > 0.05, trial p > 0.05, day p > 0.05), and neither *Jhe *nor *clt *mutant females had detectable fertility defects.

## Conclusions

### Changes in gene expression upon mating

The complex reproductive behaviors exhibited by *Drosophila *require the interaction between genetics and environment. Courtship is an innate and stereotypical process under control of the somatic sex-determination hierarchy and is influenced by social interactions. Courtship and mating elicit gene expression changes in females [49,55-57], and courtship affects transcript profiles in males [12,22]. The female post-mating effects occur rapidly (within minutes) or can be detected several hours after mating [49,55-57]. Within 5 min of courtship, whole-male gene expression profiles also change rapidly [12,22]. In this study we expanded on our earlier studies in whole males to show that courtship culminating in mating causes changes in gene expression in the male head as well. Expression levels likely change rapidly in response to sensory cues received during courtship, while the physiological changes from mating [58] may mediate long-term expression level changes in the nervous system or elsewhere in the fly that can feed back to the nervous system.

The expression profile of a 5 min courting male differs from that of a 2 hr post-mating male. This is not surprising since we expected that the process of mating would have major effects on male physiology that would be reflected in altered transcriptional profiles. Of the 47 genes with altered expression 2 hrs after mating (Tables 1 and 2), only 1 gene, *fit*, is also up regulated in males after 5 min of courtship [12]. *CG16772 *is up regulated 2 hrs after mating but is down regulated during 5 min of courtship [12]. *CG16772 *is one of several fat body-expressed immune response genes down regulated during courtship, possibly to allow energetic resources to be directed toward offspring production rather than immunity [12,22]. After mating, expression of *CG16772 *may increase because contact with a female increases the likelihood of encountering a pathogen.

The fact that few genes overlap between these data sets is not surprising since we assayed different time points (5 min or 2 hrs), different tissues (whole bodies in previous studies versus heads in this study) and different behaviors (courtship alone versus courtship culminating in mating). We also used different approaches for analyzing the data due to the differences in experimental design for each test. The analysis strategies provide us a conservative estimate of the transcripts affected by courtship and mating.

We predict that some mating-responsive genes facilitate an increased male mating efficiency for future encounters. Little is known about how repeated matings affect male mating latency, duration or fecundity. After his first mating, the male may perceive and process female stimuli more rapidly, may be more appealing to the female, or may be physiologically primed for subsequent matings by replenishment of Acps, sperm or other seminal proteins, resulting in decreased courtship or mating latencies. Alterations in gene expression, such as those described here and in our earlier work [12,22], may contribute to these expected behavioral and physiological changes.

### Gene expression in adipose tissue

The fat body is a secretory tissue [reviewed in [59]] whose effects on fly reproductive behavior have previously been described [[37,39,40,43]; reviewed in [52]]. The majority of mating-responsive genes are expressed in adult adipose tissue (fat body) (Table 4), and we analyzed a subset of six up-regulated genes to show that they are expressed in adipose tissue surrounding the brain (Figs. 2 and 3). Furthermore, we observed increased expression of *fit *in male adipose tissue after courtship followed by mating (Fig. 3). *fit *also is expressed in the head fat of females and originally was named based upon its high expression in females under the control of *Sex-lethal *[39], which is the initial regulatory gene in the somatic sex-determination hierarchy.

Other studies also indicated that several mating-responsive genes identified in our study are expressed in the fat body surrounding the brain. *Larval serum protein 2 *(*Lsp2*) is expressed in the head fat of both sexes [60]. Of the 25 genes up regulated by courtship and mating, 14 are detectable (signal strength greater than 20) in brain and 21 genes are detectable in fat body based upon a microarray analysis of adult mRNA expression levels [53]. Of these 25 up regulated genes, 16 are enriched in the fat body relative to other adult tissues (Table 4).

Taken together, these results imply that the brain is not the only tissue responding to or regulating post-mating behavior, but that adipose tissue plays a role in this process as well. In response to mating, a signaling cascade initiated by neurosecretory cells may transmit the signal to the surrounding fat body. The fat body then could perpetuate the signal by secreting factors that influence neuronal or non-neuronal tissues. We hypothesize that expression level changes in the brain alter neuronal signaling either directly or indirectly, which impacts the processing of sensory cues and targets other reproductively important tissues.

### Juvenile hormone esterases and male reproductive behavior

Another mating-responsive gene, *Jhe*, is also expressed in adipose tissue [61-64] and functions in reproductive behavior (Figs. 4, 5, 6). *Jhe *and three closely related esterase genes (*clt*, *Jhedup*, and *CG7529*) have juvenile hormone esterase (JHE) activity *in vitro*. JHEs together with juvenile hormone epoxide hydrolases hydrolyze Juvenile hormone (JH) to regulate JH levels [65,66]. Since *Jhe *expression is positively regulated by JH [67], the mating-induced increase in *Jhe *expression identified in our study may be JH dependent.

Much of our understanding of physiological functions of JH comes from studies investigating its function during development [reviewed in [68]]. However, JH also has important post-developmental functions such as promoting accessory gland protein (Acp) synthesis [69]. During mating Acps are transferred, along with sperm, to the female [70], and the transfer of Acps triggers male synthesis of new Acps [58]. Males also transfer Sex-peptide to the female during mating [71-73]. Sex-peptide increases JH levels in females [74], which stimulates egg development [75]. However, possible mating-induced changes in male JH levels have not been evaluated. Since ejaculate components must be replenished after mating, we hypothesize that male JH levels increase after mating to stimulate Acp synthesis. The increase in JH would up regulate *Jhe *expression which would, in turn, reduce JH levels once the ejaculate components have been replenished.

JH also has a role in modulating behavior since males with reduced JH court females less intensely [76], Our data suggest that an increase in JH, caused by reduction of *Jhe *or *clt*, may also disrupt courtship (Figs. 4, 5, 6). *Jhe *and *clt *deficient males, which likely have increased levels of JH, court less vigorously (Fig. 4), have increased mating latencies (Fig. 5), and have reduced mating success (Fig. 6). This situation exemplifies the complex regulation governing behavior and implies that JH levels must be tightly regulated in order to ensure appropriate behavioral and physiological responses.

### Gene expression in the brain

Although we are particularly interested in the large number of fat-expressed genes that were identified in this and earlier screens [12,22], we also note that several of the identified transcripts are expressed in brains as would be expected for genes that function in behavior. Proper function of the nervous system relies on the appropriate cellular architecture, connections and signaling. Behavior requires the sensory systems to perceive the information accurately and transmit such information to the brain for processing. The brain can then transmit the signal to the appropriate output pathways which can modify signaling in tissues such as the fat body or the brain itself. Therefore the establishment and maintenance of the brain (and sensory systems) is vital to the organism's ability to respond to its environment and experiences. It is possible that mating-responsive genes act in the development or maintenance of a mated male brain as opposed to a naïve male brain.

Thirteen of the 21 fat-expressed genes up regulated in mated males are also expressed in brains at detectable levels [53]; a single transcript, *CG4288 *is detected in brains but not fat [53]. None of these genes have known function in behavior, but their reported mutant phenotypes or molecular functions indicate that several of the loci may have important neural maintenance functions.

For example, mutants for *bgm*, an enzyme involved in fatty acid metabolism that is expressed in both the brain and fat, have a neurodegeneration phenotype in response to accumulation of long chain fatty acids [77]. Another gene that potentially functions in a neurodegeneration pathway is *Phosphatidyl-serine synthase*, which responds to changes in polygluatmate (polyQ) levels [78]. polyQ diseases, including Huntington's Disease, are adult on-set progressive neural degeneration diseases caused by the accumulation of glutamate repeats [79].

Cellular homeostasis is important in the maintenance and function of the *Drosophila* brain. One gene that helps maintain this homeostasis is *Iron regulatory protein 1B *(*Irp-1B*) which encodes a protein that binds to iron-responsive elements (IREs) to regulate iron metabolism [80]. In addition to affecting cell survival and homeostasis, neural morphology might also be regulated by mating-responsive candidates. Mutants of *Pabp2 *show pathfinding and targeting defects in the larval neuromuscular junction [81].

### Mating-responsive genes and the sex-determination hierarchy

This genome-wide analysis identified known sex-determination hierarchy target genes such as *fit*. Three other mating-responsive genes (*CG16772*, *Prx2540-2*, and *CG16898*) (Tables 1 and 2) are also regulated by the sex-determination hierarchy [41]. Transcriptional profiling of mutants for a variety of sex-determination hierarchy genes indicates that *Prx2540-2 *and *CG16898 *are regulated by *fruitless *(*fru*), while *fit *is downstream of *transformer *(*tra*). *CG16772 *may also function downstream of *tra *[41].

The splicing factor *squid *(*sqd*) is up regulated in mated male heads (Table 1). Interestingly, primary transcripts of the *sqd *locus are sex-specifically spliced in the head as well as the germline, although it is not known if *sqd *splicing is regulated by the sex-determination hierarchy [82]. It is possible that *sqd *and other mating-responsive loci function as downstream targets of the sex-determination hierarchy to regulate morphological and behavioral differences between male and female *Drosophila*. Alternatively, there may be other pathways (such as those that regulate alternative splicing) that function together with the sex-determination hierarchy to regulate reproductive behavior.

We predict that mating-responsive genes also function in other aspects of reproduction and behavior; therefore, we propose this genome-wide approach as a powerful tool for determining the genetic pathways and intracellular processes regulating reproduction, both at the behavioral and physiological levels.

## Methods

### Microarray Analysis

The wild-type *Canton-S (CS) *strain was isogenized to reduce genetic variation and the isoline was kept at 25°C on a 12-hr light/dark cycle. Twenty or fewer virgin *CS *males were aged collectively for 3 days at 25°C. On day 4, individual males were aspirated into vials. Virgin females were collected and aged in groups of 20 or fewer flies for 4 days at 25°C.

On day 5, males were equally divided into two treatment groups. One group (referred to as "mated males") consisted of individual males that were placed with a female for courtship and mating, while the second group of males (referred to as "control males") was mock exposed to a female. We tested both groups at the same time to allow for paired microarray and Cyber-T analyses (see below). For the mated male group, a single, aged virgin female was aspirated into each male's vial. The control males were treated identically except that no female was transmitted during the aspiration process.

Upon completion of mating, females were removed from the vials. Males from both treatment groups were quick frozen 2 hrs later and stored at -80°C for future RNA extraction; only pairs for which the mated male had a mating latency less than 30 min and mating duration of 18-30 min were collected for RNA extraction. Seventy-four percent of mated males tested met this requirement. All procedures were conducted at the same time each day to control for circadian effects.

Head tissue was separated from the remaining body by vortexing quick-frozen flies. Male heads were assigned to one of 20 groups (30 heads in each group; 10 mated and 10 control RNA preparations) so that control and mated samples collected together could be analyzed by paired statistical comparisons. Following standard protocols, total RNA from head tissue was extracted in Trizol (Invitrogen, Carlsbad, CA, USA). Total head RNA preparations from 10 groups (5 control groups and their corresponding mated groups) were sent to the University of Kentucky MicroArray Core Facility for labeling and hybridization to Affymetrix *Drosophila *2.0 Genome Arrays following standard Affymetrix (Santa Clara, CA, USA) protocols.

Expression values were generated similarly to previous experiments [12,22] using five algorithms (PM, PM-MM, MAS 5.0, GCRMA, and GeneSpring). Multiple expression value algorithms were used to control for variation among the algorithms and to generate a statistically stronger candidate gene set. We used dChip's PM (perfect match between the probe and target sequence) and PM-MM (one nucleotide between the probe and target sequence is mismatched) algorithms [83], as well as those implemented by GCOS (MAS 5.0, Affymetrix), R (GCRMA) [84], and GeneSpring (Agilent, Santa Clara, CA, USA). For the dChip algorithms, expression values were only considered if greater than 50; for the other 3 methods, expression values were required to be greater than 100. To test for significance, we used Cyber-T's Bayesian *t*-test analysis [85]. Candidate mating-responsive genes included those whose expression differed significantly (p < 0.001) between control male heads and mated male heads in at least 3 expression value data sets and had a false discovery rate less than 0.05 [86]. With such stringent criteria, we did not specify a particular fold change cut-off value.

### qPCR

To confirm the microarray results, qPCR was performed on 10 independent samples (5 mated and 5 control RNA preparations) that were collected as described above but were not used in the microarray analysis. polyA^+ ^RNA was isolated from each of the 10 samples using the Oligotex mRNA mini kit (Qiagen, Netherlands). cDNA was synthesized using the SuperScript First-Strand Synthesis System (Invitrogen, Carlsbad, CA, USA). We designed primers to amplify 10 up-regulated and 3 down-regulated genes, choosing genes that are predicted to be enriched in brain, fat body or both tissues based upon FlyAtlas expression data [53]. When possible, primer pairs were designed across introns to control for amplification specificity. Genes that are expressed at low levels in the head [[53]; L. L. Ellis and G. E. Carney, unpublished results] were not tested.

Using the SYBR Green PCR Mastermix (Applied Biosystems, Foster City, CA, USA), 2 μL of a 1:4 dilution of each template was run in triplicate in the ABI7500 (Applied Biosystems, Foster City, CA, USA) using default parameters. Control reactions lacking template and controls with template but without Reverse Transcriptase were used. Primer-specific amplification was determined by analyzing dissociation curves for each primer pair.

mRNA levels were determined by the Relative Standard Curve Method (Applied Biosystems, Foster City, CA, USA), and candidate gene transcript levels were normalized to *rp49 *transcript levels. Normalizing the mated male transcript levels to the control male transcript levels generated a relative fold change. We also analyzed trends in the average relative transcript levels of each treatment (control and mated) using the two-tailed *t*-test. Secondary qPCR analysis confirmed increased expression of *CG6188 *and decreased expression of *alpha Esterase-2*.

Regression of mean expression microarray analysis fold changes compared to independent qPCR fold changes indicated a highly significant positive correlation between results obtained by the two methods (r = 0.51, N = 10, p = 0.021).

### *In situ *hybridization

Digoxigenin (DIG)-labeled sense and antisense RNA probes were made from cDNA clones for six candidate genes with predicted fat body expression following the manufacturer's standard protocol (Roche, Nutley, NJ, USA). The genes and their corresponding cDNA clones were *CG4825 *(LD10327), *CG8449 *(GH10459), *CG13360 *(LP09811), *bubblegum *(*bgm*) (GM14009), *fit *(RH40291) and *Prx2540-2 *(RH69586). Expression of *fit *is regulated by *tra *while expression of *Prx2540-2 *is regulated by *fruitless *(*fru*) [41]; *tra *and *fru *are regulatory components of the sex-determination hierarchy. Antisense and sense probes were hydrolyzed into 200 bp fragments and *in situ *hybridization to male brains, head carcass and abdominal cuticle was performed as described in [33]. Antisense probes detected expressed transcripts in each case, while sense probes served as negative controls for expression.

To verify the increased expression of *fit *in male head tissue after courtship followed by mating, virgin *CS *males were collected 2 hrs after mating and compared to virgin *CS *control males that did not mate with a female. After treatment, males were cryosectioned in OCT compound and *in situ *hybridization was performed on the sections as described previously [37]. Control and mated tissues were placed on the same slide to control for histochemical reaction time. We qualitatively assessed *fit *expression in adipose tissue lining the brain from non-existent (-) to highly expressed (+++).

### Courtship assays

All flies were kept on a 12-hr light/dark cycle at 25°C. P-element insertion mutations in *Jhe *and *cricklet *(*clt*) were obtained from the Bloomington *Drosophila* Stock Center (*clt^BG01317^*) and the Exelixis Collection at Harvard Medical School (*Jhe^e01859^*). These insertions are likely hypomorphs since they are located in proximal promoter regions. Each P-element was backcrossed into the *CS *background to generate a genetically similar control that had one wild-type copy of *Jhe *or *clt*. To test for a genetic interaction between *Jhe *and *clt*, the two insertion strains, *Jhe^e01859 ^*and *clt^BG01317^*, were crossed to generate transheterozygous flies containing a single P-element insertion in each gene (*Jhe^e01859 ^+/+ clt^BG01317^*). Virgin P-insertion or control males were collected and stored individually for 4 to 5 days; virgin *CS *females were aged collectively for 3 to 5 days.

Behavioral assays were conducted at 22°C under red light conditions to diminish the effect of eye color on vision and courtship. We video recorded the interactions with a digital camcorder so that subsequent analyses could be performed. To analyze courtship behavior, a male was aspirated into a mating chamber (diameter = 1 cm) and a virgin *CS *female was introduced 2 min later. The pair was video recorded for 10 min. The courtship index (CI; percentage of time the male spent performing courtship during the initial 10 min of observation) and courtship latency (time until courtship occurs) were calculated. CI values were arcsine transformed for statistical analysis. Two-tailed *t*-test comparisons between homozygous mutants and controls were calculated to determine significance (p < 0.05). *Jhe^e01859 ^+/+ clt^BG01317 ^*males were compared to both control genotypes (two-tailed *t*-test).

### Fertility Assays

The ability of a male to mate with multiple *CS *females and the fecundity of these matings was also assessed. *Jhe *and *clt *mutants and heterozygous controls, as well as *CS *virgin females, were collected and aged as described for the courtship assay. Under red light, a male was aspirated into a mating chamber, followed by a *CS *virgin female. The male was given 2 hrs to mate with the female. If mating occurred, the female was placed in a vial with food to measure fecundity (number of eggs laid and number of adult offspring) and the male was placed in a new mating chamber. A second *CS *virgin female was aspirated into the new chamber and the pair was given 2 hrs to mate. If the second mating occurred, the female was placed in a vial for later progeny counts, and the male was moved to another chamber for mating with a third and final female. The third mated female was also kept for further analysis. For the first mating bout, all 10 c*lt^BG01317 ^*males mated, while only three of the ten males mated with the second female and none of the 3 males mated with the third female. Eight out of 13 *clt^BG01317/^+ *males mated with the first female, five of those eight males mated with the second female and four of the remaining five males mated with the third female. *Jhe^e01859 ^*males only mated with the first female (six out of nine males). However, nine of ten *Jhe^e01859^/*+ males mated with the first female, seven of those nine males mated with the second female and four of the seven males mated with the third female. For the transheterozygous *clt^BG01317^/Jhe^e01859 ^*males, seven of 12 mated with the first female, four of seven males mated with the second female and two of the four males mated with the third female.

The mating latencies and durations for each of the three possible matings were measured and significance was determined by Univariate ANOVA analysis using genotype and mating trial as fixed variables with Tukey's post-hoc analysis (SPSS). Males that did not mate within the 2 hr window were scored as being unsuccessful. Using linear regression, we assessed the significance (p < 0.05) of genotype and mating bout on mating success.

For 6 days following the assay, the female was transferred to a new vial and the number of eggs laid in each vial was determined. Vials were maintained at 25°C for 18 days to allow for a count of the total number of adult progeny. Significant effects of genotype and trial on mating latency or duration were measured by the Univariate ANOVA and Tukey's post-hoc analysis. We also measured the significance of genotype, mating bout and day of egg laying on the male's fecundity (Univariate ANOVA and Tukey's post-hoc analysis). Fecundity was measured by the total number of eggs laid and by the arcsine transformed ratio of adult offspring to eggs laid.

## Authors' contributions

LLE designed and executed experiments, analyzed the data, and helped write the manuscript. GEC conceived and designed experiments and helped write the manuscript. All authors read and approved the final draft.

## References

[B1] Dukas R Mooers AO Environmental enrichment improves mating success in fruit flies Anim Behav 2003 66 741 749 10.1006/anbe.2002.2261

[B2] Gailey DA Hall JC Siegel RW Reduced reproductive success for a conditioning mutant in experimental populations of *Drosophila melanogaster* Genetics 1985 111 795 804 3934032 10.1093/genetics/111.4.795 PMC1202672

[B3] Siegel RW Hall JC Conditioned responses in courtship behavior of normal and mutant Drosophila Proc Natl Acad Sci USA 1979 76 3430 3434 10.1073/pnas.76.7.3430 16592682 PMC383839

[B4] Ueda A Kidokoro Y Aggressive behaviors of female *Drosophila melanogaster *are influenced by their social experience and food resources Physiol Entomol 2002 27 21 28 10.1046/j.1365-3032.2002.00262.x

[B5] Yurkovic A Wang O Basu AC Kravitz EA Learning and memory associated with aggression in *Drosophila melanogaster* Proc Natl Acad Sci USA 2006 103 17519 17524 10.1073/pnas.0608211103 17088536 PMC1634832

[B6] Kozorovitskiy Y Hughes M Lee K Gould E Fatherhood affects dendritic spines and vasopressin V1a receptors in the primate prefrontal cortex Nat Neurosci 2006 9 1094 1095 10.1038/nn1753 16921371

[B7] Mori T Hiraka I Kurata Y Kawachi H Kishida O Nishimura K Genetic basis of phenotypic plasticity for predator-induced morphological defenses in anuran tadpole, *Rana pirica*, using cDNA subtraction and microarray analysis Biochem Biophys Res Commun 2005 330 1138 1145 10.1016/j.bbrc.2005.03.091 15823562

[B8] Stewart BA McLean JR Population density regulates Drosophila synaptic morphology in a Fasciclin-II-dependent manner J Neurobiol 2004 61 392 399 10.1002/neu.20096 15490479

[B9] Technau GM Fiber number in the mushroom bodies of adult *Drosophila melanogaster *depends on age, sex, and experience J Neurogenet 2007 21 183 196 10.1080/01677060701695359 18161582

[B10] Anseloni VCZ He F Novikova SI Robbins MT Lidow IA Ennis M Lidow MS Alterations in stress-associated behaviors and neurochemical markers in adult rats after neonatal short-lasting local inflammatory insult Neuroscience 2005 131 635 645 10.1016/j.neuroscience.2004.11.039 15730869

[B11] Burmeister SS Jarvis ED Fernald RD Rapid behavioral and genomic responses to social opportunity PLoS Biol 2005 3 1996 2004 10.1371/journal.pbio.0030363 PMC1255743 16216088

[B12] Carney GE A rapid genome-wide response to *Drosophila melanogaster *social interactions BMC Genomics 2007 8 288 10.1186/1471-2164-8-288 17714588 PMC1999498

[B13] Levine JD Funes P Dowse HB Hall JC Resetting the circadian clock by social experience in *Drosophila melanogaster* Science 2002 298 2010 2012 10.1126/science.1076008 12471264

[B14] Mehren JE Griffith LC Calcium-independent calcium/calmodulin-dependent protein kinase II in the adult Drosophila CNS enhances the training of pheromonal cues J Neurosci 2004 24 10584 10593 10.1523/JNEUROSCI.3560-04.2004 15564574 PMC6730130

[B15] Murata S Yoshiara T Lim CR Sugino M Kogure M Ohnuki T Komurasaki T Matsubara K Psychophysiological stress-regulated gene expression in mice FEBS Lett 2005 579 2137 2142 10.1016/j.febslet.2005.02.069 15811331

[B16] Reiser M Poeggel G Schnabel R Schroder H Braun K Effect of social experience on dopamine-stimulated adenylyl cyclase activity and G protein composition in chick forebrain J Neurochem 1999 73 1293 1299 10.1046/j.1471-4159.1999.0731293.x 10461924

[B17] Shen CP Tsimberg Y Salvadore C Meller E Activation of Erk and JNK MAPK pathways by acute swim stress in rat brain regions BMC Neurosci 2004 5 36 10.1186/1471-2202-5-36 15380027 PMC526203

[B18] Greenspan RJ Understanding the genetic construction of behavior Sci Am 1995 272 72 78 10.1038/scientificamerican0495-72 7716488

[B19] Greenspan RJ Ferveur J-F Courtship in Drosophila Annu Rev Genet 2000 34 205 232 10.1146/annurev.genet.34.1.205 11092827

[B20] Billeter J-C Goodwin SF O'Dell KM Genes mediating sex-specific behaviors in Drosophila Adv Genet 2002 47 87 116 full_text 12000098 10.1016/s0065-2660(02)47003-4

[B21] Tompkins L Genetic analysis of sex appeal in Drosophila Behav Genet 1984 14 411 440 10.1007/BF01065443 6441562

[B22] Ellis LL Carney GE *Drosophila melanogaster *males respond differently at the behavioral and genome-wide levels to *Drosophila melanogaster *and *Drosophila simulans *females J Evol Biol 2009 22 2183 2191 10.1111/j.1420-9101.2009.01834.x 19765174

[B23] Siwicki KK Ladewski L Associative learning and memory in Drosophila: beyond olfactory conditioning Behav Processes 2003 64 225 238 10.1016/S0376-6357(03)00137-2 14556954

[B24] Ewing AW Functional-aspects of Drosophila courtship Biol Rev 1983 58 275 292 10.1111/j.1469-185X.1983.tb00390.x

[B25] Mehren JE Ejima A Griffith LC Unconventional sex: Fresh approaches to courtship learning Curr Opin Neurobiol 2004 14 745 750 10.1016/j.conb.2004.10.012 15582378

[B26] Cline TW Reflections on a path to sexual commitment Genetics 2005 169 1179 1185 15795241 10.1093/genetics/169.3.1179 PMC1449525

[B27] Shirangi TR McKeown M Sex in flies: What 'body-mind' dichotomy? Dev Biol 2007 306 10 19 10.1016/j.ydbio.2007.03.022 17475234

[B28] Demir E Dickson BJ *fruitless *splicing specifies male courtship behavior in Drosophila Cell 2005 121 785 794 10.1016/j.cell.2005.04.027 15935764

[B29] Finley KD Taylor BJ Milstein M McKeown M *dissatisfaction*, a gene involved in sex-specific behavior and neural development of *Drosophila melanogaster* Proc Natl Acad Sci USA 1997 94 913 918 10.1073/pnas.94.3.913 9023356 PMC19613

[B30] Kimura K Ote M Tazawa T Yamamoto D Fruitless specifies sexually dimorphic neural circuitry in the Drosophila brain Nature 2005 438 229 233 10.1038/nature04229 16281036

[B31] Manoli DS Foss M Villella A Taylor BJ Hall JC Baker BS Male-specific *fruitless *specifies the neural substrates of Drosophila courtship behavior Nature 2005 436 395 400 15959468 10.1038/nature03859

[B32] Stockinger P Kvitsiani D Rotkopf S Tirian L Dickson BJ Neural circuitry that governs Drosophila male courtship behavior Cell 2005 121 795 807 10.1016/j.cell.2005.04.026 15935765

[B33] Arbeitman MN Fleming AA Siegal ML Null BH Baker BS A genomic analysis of Drosophila somatic sexual differentation and its regulation Development 2004 131 2007 2021 10.1242/dev.01077 15056610

[B34] Burtis KC Coschigano KT Baker BS Wensink PC The Doublesex proteins of *Drosophila melanogaster *bind directly to a sex-specific yolk protein gene enhancer EMBO J 1991 10 2577 2582 1907913 10.1002/j.1460-2075.1991.tb07798.x PMC452955

[B35] Cann MJ Chung E Levin LR A new family of adenylyl cyclase genes in the male germline of *Drosophila melanogaster* Dev Genes Evol 2000 210 200 206 10.1007/s004270050304 11180822

[B36] Dalton JE Lebo MS Sanders LE Sun F Arbeitman MN Ecdysone receptor acts in *fruitless- *expressing neurons to mediate *Drosophila *courtship behaviors Curr Biol 2009 19 1447 1452 10.1016/j.cub.2009.06.063 19646872 PMC2763606

[B37] Dauwalder B Tsujimoto S Moss J Mattox W The Drosophila *takeout *gene is regulated by the somatic sex-determination pathway and affects male courtship behavior Genes Dev 2002 16 2879 2892 10.1101/gad.1010302 12435630 PMC187483

[B38] Drapeau MD Radovic A Wittkopp PJ Long AD A gene necessary for normal male courtship, *yellow*, acts downstream of *fruitless *in the *Drosophila melanogaster *larval brain J Neurobiol 2003 55 53 72 10.1002/neu.10196 12605459

[B39] Fujii S Amrein H Genes expressed in the Drosophila head reveal a role for fat cells in sex-specific physiology EMBO J 2002 21 5353 5363 10.1093/emboj/cdf556 12374736 PMC129088

[B40] Fujii S Toyama A Amrein H A male-specific fatty acid omega-hydroxylase, SXE1, is necessary for efficient male mating in *Drosophila melanogaster* Genetics 2008 180 179 90 10.1534/genetics.108.089177 18716335 PMC2535673

[B41] Goldman TD Arbeitman MN Genomic and functional studies of Drosophila sex hierarchy regulated gene expression in adult head and nervous system tissues PLoS Genet 2007 3 e216 10.1371/journal.pgen.0030216 18039034 PMC2082469

[B42] Kopp A Duncan I Godt D Carroll SB Genetic control and evolution of sexually dimorphic characters in Drosophila Nature 2000 408 553 559 10.1038/35046017 11117736

[B43] Lazareva AA Roman G Mattox W Hardin PE Dauwalder B A role for the adult fat body in Drosophila male courtship behavior PLoS Genet 2007 3 e16 10.1371/journal.pgen.0030016 17257054 PMC1781494

[B44] Dukas R Experience improves courtship in male fruit flies Anim Behav 2005 69 1203 1209 10.1016/j.anbehav.2004.08.012

[B45] Polejack A Tidon R Learning of courtship components in *Drosophila mercatorum *(Paterson & Wheller) (Diptera, Drosophilidae) Rev Bras Entomol 2007 51 82 86 10.1590/S0085-56262007000100014

[B46] Anholt RRH Dilda CL Chang S Fanara JJ Kulkarni NH Ganguly I Rollmann SM Kamdar KP Mackay TFC The genetic architecture of odor-guided behavior in Drosophila: Epistasis and the transcriptome Nat Genet 2003 35 180 184 10.1038/ng1240 12958599

[B47] Ceriani MF Hogenesch JB Yanovsky M Panda S Straume M Kay SA Genome-wide expression analysis in Drosophila reveals genes controlling circadian behavior J Neurosci 2002 22 9305 9319 12417656 10.1523/JNEUROSCI.22-21-09305.2002 PMC6758054

[B48] Dubnau J Chiang AS Grady L Barditch J Gossweiler S McNeil J Smith P Buldoc F Scott R Certa U Broger C Tully T The *staufen/pumilio *pathway is involved in Drosophila long-term memory Curr Biol 2003 13 286 296 10.1016/S0960-9822(03)00064-2 12593794

[B49] Lawniczak MK Begun DJ A genome-wide analysis of courting and mating responses in *Drosophila melanogaster *females Genome 2004 47 900 910 10.1139/g04-050 15499404

[B50] Mackay TFC Heinsohn SL Lyman RF Moehring AJ Morgan TJ Rollmann SM Genetics and genomics of Drosophila mating behavior Proc Natl Acad Sci USA 2005 102 6622 6629 10.1073/pnas.0501986102 15851659 PMC1131870

[B51] Toma DP White KP Hirsch J Greenspan RJ Identification of genes involved in *Drosophila melanogaster *geotaxis, a complex behavioral trait Nat Genet 2002 31 349 353 12042820 10.1038/ng893

[B52] Dauwalder B Systems behavior: Of male courtship, the nervous system and beyond in Drosophila Curr Genomics 2008 9 517 524 10.2174/138920208786847980 19516958 PMC2694563

[B53] Chintapalli VR Wang J Dow JA Using FlyAtlas to identify better *Drosophila melanogaster *models of human disease Nat Genet 2007 39 715 720 10.1038/ng2049 17534367

[B54] Campbell PM Harcourt RL Crone EJ Claudianos C Hammock BD Russell RJ Oakeshott JG Identification of a juvenile hormone esterase gene by matching its peptide mass fingerprint with a sequence from the Drosophila genome project Inesct Biochem Mol Biol 2001 31 513 520 10.1016/S0965-1748(01)00035-2 11267890

[B55] Mack PD Kapelnikov A Heifetz Y Bender M Mating-responsive genes in reproductive tissues of female *Drosophila melanogaster* Proc Natl Acad Sci USA 2006 103 10358 10363 10.1073/pnas.0604046103 16798875 PMC1502462

[B56] McGraw LA Clark AG Wolfner MF Post-mating gene expression profiles of female *Drosophila melanogaster *in response to time and to four male accessory gland proteins Genetics 2008 179 1395 1408 10.1534/genetics.108.086934 18562649 PMC2475742

[B57] McGraw LA Gibson G Clark AG Wolfner MF Genes regulated by mating, sperm, or seminal proteins in mated female *Drosophila melanogaster* Curr Biol 2004 14 1509 1514 10.1016/j.cub.2004.08.028 15324670

[B58] DiBenedetto AJ Harada HA Wolfner MF Structure, cell-specific expression, and mating-induced regulation of a *Drosophila melanogaster *male accessory gland gene Dev Biol 1990 139 134 148 10.1016/0012-1606(90)90284-P 2109712

[B59] Schlegel A Stainier DYR Lessons from "lower" organisms: What worms, flies, and zebrafish can teach us about human energy metabolism PLoS Genet 2007 3 2037 2048 10.1371/journal.pgen.0030199 PMC2098794 18081423

[B60] Benes H Edmondson RG Fink P Keizlarova-Lepesant J Lepesant JA Miles JP Spivey DW Adult expression of the Drosophila *Lsp-2 *gene Dev Biol 1990 142 138 146 10.1016/0012-1606(90)90157-E 2227091

[B61] Anand A Crone EJ Zera AJ Tissue and stage-specific juvenile hormone (JHE) and epoxide hydrolase (JHEH) enzyme activities and *Jhe *transcript abundance in lines of the cricket *Gryllus assimilis *artificially selected for plasma JHE activity: Implications for JHE microevolution J Insect Physiol 2008 54 1323 1331 10.1016/j.jinsphys.2008.06.006 18634793

[B62] Kamimura M Takahashi M Kikuchi K Reza AMS Kiuchi M Tissue-specific regulation of *Juvenile hormone esterase *gene expression by 20-hydroxyecdysone and Juvenile hormone in *Bombyx mori* Arch Insect Biochem 2007 65 143 151 10.1002/arch.20186 17570489

[B63] Klages G Emmerich H Juvenile hormone metabolism and juvenile hormone esterase titer in hemolymph and peripheral tissues of *Drosophila hydei* J Comp Physiol 1979 132 319 325

[B64] Shanmugavelu S Baytan AR Chesnut JD Bonning BC A novel protein that binds juvenile hormone esterase in fat body tissue and pericardial cells of the tobacco hornworm *Manduca sexta* J Biol Chem 2000 275 1802 1806 10.1074/jbc.275.3.1802 10636878

[B65] Campbell PM Healy MJ Oakeshott JG Characterization of *juvenile hormone esterase *in *Drosophila melanogaster* Insect Biochem Mol Biol 1992 22 665 677 10.1016/0965-1748(92)90045-G 9718682

[B66] Campbell PM Oakeshott JG Healy MJ Purification and kinetic characterization of *juvenile hormone esterase *from *Drosophila melanogaster* Insect Biochem Mol Biol 1998 28 501 515 10.1016/S0965-1748(98)00037-X 9718682

[B67] Kethidi DR Xi ZY Palli SR Developmental and hormonal regulation of *juvenile hormone esterase *gene in *Drosophila melanogaster* J Insect Physiol 2005 51 393 400 10.1016/j.jinsphys.2004.12.007 15890182

[B68] Flatt T Tu MP Tatar M Hormonal pleiotropy and the juvenile hormone regulation of Drosophila development and life history Bioessays 2005 27 999 1010 10.1002/bies.20290 16163709

[B69] Wolfner MF Partridge L Lewin S Kalb JM Chapman T Herndon LA Mating and hormonal triggers regulate accessory gland gene expression in male Drosophila J Insect Physiol 1997 43 1117 1123 10.1016/S0022-1910(97)00062-0 12770484

[B70] Wolfner MF Tokens of love: Functions and regulation of Drosophila male accessory gland products Insect Biochem Mol Biol 1997 27 179 192 10.1016/S0965-1748(96)00084-7 9090115

[B71] Chen PS The accessory gland proteins in male Drosophila: Structural, reproductive, and evolutionary aspects Experientia 1996 52 503 510 10.1007/BF01969718 8698082

[B72] Kubli E The sex-peptide Bioessays 1992 14 779 784 10.1002/bies.950141111 1365892

[B73] Wolfner MF Harada HA Bertram MJ Stelick TJ Kraus KW Kalb JM Lung YO Neubaum DM Park M Tram U New genes for male accessory gland proteins in *Drosophila melanogaster* Insect Biochem Mol Biol 1997 27 825 834 10.1016/S0965-1748(97)00056-8 9474779

[B74] Moshitzky P Fleischmann I Chaimov N Saudan P Klauser S Kubli E Applebaum SW Sex-peptide activates juvenile hormone biosynthesis in the *Drosophila melanogaster *corpus allatum Arch Insect Biochem Physiol 1996 32 363 374 10.1002/(SICI)1520-6327(1996)32:3/4<363::AID-ARCH9>3.0.CO;2-T 8756302

[B75] Soller M Bownes M Kubli E Control of oocyte maturation in sexually mature Drosophila females Dev Biol 1999 208 337 351 10.1006/dbio.1999.9210 10191049

[B76] Wilson TG DeMoor S Lei J Juvenile hormone involvement in *Drosophila melanogaster *male reproduction as suggested by the methoprene-tolerant(27) mutant phenotype Insect Biochem Mol Biol 2003 33 1167 1175 10.1016/j.ibmb.2003.06.007 14599489

[B77] Min KT Benzer S Preventing neurodegeneration in the Drosophila mutant *bubblegum* Science 1999 248 1958 10.1126/science.284.5422.1985 10373116

[B78] Nelson B Nishimura S Kanuka H Kuranaga E Inoue M Hori G Nakahara H Miura M Isolation of gene sets affected specifically by polyglutamine expression: Implication of the TOR signaling pathway in neurodegeneration Cell Death Diffn 2005 12 1115 1123 10.1038/sj.cdd.4401635 15861189

[B79] Zoghbi HY Orr HT Glutamine repeats and neurodegeneration Ann Rev Neurosci 2000 23 217 247 10.1146/annurev.neuro.23.1.217 10845064

[B80] Muckenthaler M Gunkel N Frishman D Cyrklaff A Tomancak P Hentze MW Iron-regulatory protein-1 (IRP-1) is highly conserved in two invertebrate species-characterization of IRP-1 homologues in *Drosophila melanogaster *and *Caenorhabditis elegans* Europ J Biochem 1998 254 230 237 10.1046/j.1432-1327.1998.2540230.x 9660175

[B81] Liebl FLW Werner KM Sheng Q Karr JE McCabe BD Featherstone DE Genome-wide P-element screen for Drosophila synaptogenesis mutants J Neurobiol 2006 66 332 347 10.1002/neu.20229 16408305 PMC1626350

[B82] Telonis-Scott M Kopp A Wayne ML Nuzhdin SV McIntyre LM Sex-specific splicing in Drosophila: Widespread occurrence, tissue specificity and evolutionary conservation Genetics 2009 181 421 434 10.1534/genetics.108.096743 19015538 PMC2644937

[B83] Li C Wong WH Model-based analysis of oligonucleotide arrays: Expression index computation and outlier detection Proc Natl Acad Sci USA 2001 98 31 36 10.1073/pnas.011404098 11134512 PMC14539

[B84] R Development Core Team R: A language and environment for statistical computing 2008 R Foundation for Statistical Computing, Vienna

[B85] Baldi P Long AD A bayesian framework for the analysis of microarray expression data: Regularized *t*-test and statistical inferences of gene changes Bioinformatics 2001 17 509 519 10.1093/bioinformatics/17.6.509 11395427

[B86] Storey JD Tibshiriani R Statistical significance for genomewide studies Proc Natl Acad Sci USA 2003 100 9440 9445 10.1073/pnas.1530509100 12883005 PMC170937

